# Beyond Lipidation: CSF APOE4 Protein Burden, Not HDL Subclass, Drives Tau Associations in APOE4 Alzheimer’s Disease

**DOI:** 10.21203/rs.3.rs-9349576/v1

**Published:** 2026-04-24

**Authors:** Zoe E. Tsokolas, Isaac Asante, Amaryllis A. Tsiknia, Juan Pablo Barbosa-Carvajal, Lailla Burka, Abhay Sagare, Gloria Patricia Cardona-Gomez, Wendy J. Mack, Meredith N. Braskie, Dobrin Nedelkov, Ronald M. Krauss, Hussein N. Yassine

**Affiliations:** University of Southern California; University of Southern California; University of Southern California; Universidad de Antioquia; University of Southern California; University of Southern California; Universidad de Antioquia; University of Southern California; University of Southern California; Isoformix Inc.; University of California San Francisco; University of Southern California

**Keywords:** APOE isoforms, brain-peripheral APOE4, HDL, Alzheimer’s

## Abstract

Apolipoprotein E ε4 (APOE4) is the strongest genetic risk factor for late-onset Alzheimer’s disease (AD), yet whether its pathogenic effects are driven by HDL lipidation state or by elevated APOE4 levels in the CNS remains unclear. Using ADNI data, we analyzed cerebrospinal fluid (CSF) and plasma small and large HDL particle concentrations, total APOE levels, and isoform-specific APOE3 and APOE4 protein levels in 144 participants with APOE ε3/ε3, ε3/ε4, or ε4/ε4 genotypes, grouped by cognitive state and memory progression over 4 years. Memory loss was defined as ≥ 10% decline on the Rey Auditory Verbal Learning Test (RAVLT) delayed recall between baseline and 48-month follow-up. Cross-sectional associations with CSF Aβ1-42, total tau, and p-tau181 were evaluated using covariate-adjusted linear regression, and longitudinal trajectories were examined using linear mixed-effects models over 6 years. CSF small and large HDL levels were higher in cognitively normal individuals and non-progressors, while APOE4 carriers exhibited reduced CSF small HDL relative to ε3 homozygotes. Importantly, APOE4 status moderated HDL–biomarker associations in opposing directions: in carriers, higher CSF small HDL was associated with lower tau and Aβ1–42, whereas in non-carriers, higher CSF small HDL was associated with higher CSF tau levels. Higher CSF APOE4 protein levels were associated with elevated tau and p-tau181, while plasma APOE measures showed minimal and often opposing associations with CSF biomarkers. Longitudinally, higher baseline CSF APOE4 proportion was associated with greater memory decline over 6 years but did not predict biomarker change. These findings argue against a protective role of APOE4 lipidation in AD and instead support a CNS-compartment model in which APOE4 protein burden aligns more closely with tau pathology and cognitive decline than peripheral APOE or HDL measures.

## Introduction

Alzheimer’s disease (AD) is characterized by altered lipid metabolism in which Apolipoprotein E (APOE) isoform-specific differences are thought to impair Aβ clearance. APOE is the major lipid transport protein in the brain, responsible for maintaining neuronal and vascular health through cholesterol delivery and membrane integrity (1). The APOE-ε4 allele confers an altered protein conformation that reduces lipid transport efficiency and disrupts lipid homeostasis, heightening neuronal vulnerability and promoting both vascular risk and neuro-inflammation (1). Carriers of the APOE-ε4 allele additionally show increased amyloid deposition, tau phosphorylation, and accelerated cognitive decline relative to non-carriers (2, 3). Despite this strong genetic association, the mechanism by which APOE4 confers AD risk in humans remains unresolved. In particular, it is unclear whether APOE4-associated pathology is driven by altered lipoprotein state or by increased APOE4 protein levels within the central nervous system (CNS).

Understanding how APOE4 and its associated HDL particles contribute to AD requires separate evaluation of central and peripheral compartments, as APOE in plasma and cerebrospinal fluid (CSF) may differentially influence lipid homeostasis and Aβ metabolism. Because the blood–brain and blood–CSF barriers restrict lipoprotein exchange, APOE exists in biologically distinct pools, with CSF APOE synthesized largely within the CNS rather than derived from peripheral sources (4, 5). In the CSF, APOE is carried on HDL-like particles that support ABCA1-mediated lipid efflux, cholesterol transport, and Aβ trafficking, functions critical for CNS homeostasis. In plasma, HDL mediates reverse cholesterol transport from peripheral tissues to the liver (6). These HDL particles are heterogeneous, comprising small and large subspecies that differ in composition, function, and tissue distribution, and whose relative abundance in CSF may influence both local and systemic lipid homeostasis (7). Whether peripheral HDL alterations reflect central APOE biology or whether CNS APOE operates independently of plasma HDL dynamics remains uncertain (8), and it is unclear whether APOE4-associated risk is mediated primarily through central (CSF) or peripheral (plasma) pathways.

Recent studies suggest that APOE4 carriers exhibit altered HDL particle composition and poor lipidation, leading to the hypothesis that reduced APOE lipidation contributes to toxicity (9). Independent studies have shown that small HDL particles in the CSF, but not HDL measures in plasma, correlate with AD-related biomarkers and cognitive performance (7). These observations have motivated therapeutic strategies aimed at enhancing APOE lipidation or increasing HDL-associated APOE. Yet direct human evidence testing whether higher HDL subclass abundance mitigates APOE4-associated biomarker abnormalities is lacking. Moreover, few studies have directly quantified isoform-specific APOE protein levels in both CSF and plasma to determine whether APOE4 protein burden itself, rather than HDL subclass distribution, aligns more closely with AD pathology. We fill this gap in the literature by quantifying isoform-specific APOE concentrations in addition to CSF HDL subclasses to examine whether these differentially associate with AD biomarker levels as a function of APOE4 status.

In the present study, we address these gaps by integrating CSF and plasma HDL particle measurements with total and isoform-specific APOE protein quantification in a well-characterized cohort of older adults with normal cognition or mild cognitive impairment from the Alzheimer’s Disease Neuroimaging Initiative (ADNI). We evaluate cross-sectional associations between CSF HDL subclasses, isoform-specific APOE concentrations, and established AD biomarkers (Aβ1–42, total tau, p-tau181) measured in CSF. We further examine whether quantitative CNS APOE4 burden predicts longitudinal cognitive decline over six years independent of genotype status. By directly comparing central and peripheral APOE measures and testing whether HDL subclass abundance modifies APOE4-associated biomarker relationships, this study tests whether APOE4-associated pathology is better explained by a lipidation-deficiency model or by a CNS APOE4 protein burden model.

## Methods

Data used in the preparation of this article were obtained from the Alzheimer’s Disease Neuroimaging Initiative (ADNI) database (adni.loni.usc.edu). The ADNI was launched in 2003 as a public-private partnership, led by Principal Investigator Michael W. Weiner, MD. The primary goal of ADNI has been to test whether serial magnetic resonance imaging (MRI), positron emission tomography (PET), other biological markers, and clinical and neuropsychological assessment can be combined to measure the progression of mild cognitive impairment (MCI) and early Alzheimer’s disease (AD).

### Human Ethics and Consent to Participate Declaration

ADNI adheres to ethical standards that include the Declaration of Helsinki, the International Conference on Harmonization Good Clinical Practice (ICH GCP), and the Code of Federal Regulations (CFR). ADNI also obtains informed consent from participants and follows the ethical standards of the institutional review board (IRB).

### Participants

The present study analyzed data from 144 participants (mean age 73.0 ± 6.6) from the ADNI dataset (Table 1). Participants with complete baseline measurements of CSF and plasma small and large HDL particle levels, CSF biomarker levels (tau, p-tau181, Aβ1–42), complete measurements of CSF and plasma total APOE, and with available Rey Auditory Verbal Learning Test (RAVLT) scores were included in our study (n = 144), with exclusions for ε2/ε3 genotype (n = 11). Measures of CSF and plasma APOE3 and APOE4 protein levels were available for 85 of the 144 participants in our sample. Individuals were stratified into four clinical groups based on delayed recall test performance (RAVLT) following methods previously described (10): (1) cognitively normal (CN) stable (n = 27), with no cognitive impairment at baseline assessment and < 10% RAVLT decline between baseline and 48-month follow-up; (2) CN decline (n = 38), with no cognitive impairment at baseline but > 10% RAVLT decline between baseline assessment and 48-months follow-up; (3) Late Mild Cognitive Impairment (LMCI) stable (n = 37), with participants having mild cognitive impairment (MCI) at baseline but no AD progression between baseline and 48-months of follow-up; lastly (4), LMCI decline (n = 42) with participants having MCI at baseline and AD progression between baseline and 48-months of follow-up.

### Study data

Biomarker data were available from CSF samples collected during annual visits spanning a 6-year period. The concentrations of Aβ1–42, t-tau, and p-tau181 in the CSF were measured using the Luminex platform INNOBIA AlzBio3 RUO test, using a microbead-based multiplex immunoassay as previously described (11). The CSF Aβ1–42 assay had a range of 200 to 1700 pg/mL (Elecsys CSF Aβ42 immunoassay) and samples with Aβ42 levels that exceeded this range were assigned the maximum value of 1700 pg/mL (n = 27) (12). For cognitive measures, the RAVLT accuracy score of delayed memory recall by % forgotten on a list-recall paradigm was used (13). RAVLT scores, used for clinical group categorization, were used in cognitive analyses and were available from each annual visit over the 6-year period.

### Total APOE Measurement

Cerebrospinal fluid (CSF) and plasma levels of APOE were determined using a Meso Scale Discovery (MSD) R-PLEX human APOE singleplex assay (Catalog number K151AMLR) following the manufacturer’s standard recommendations for assay protocol 1. Briefly, 25 μl per well of biotinylated capture antibody diluted in coating diluent 100 (Catalog number R50AA, MSD) was added to each well of MSD small spot streptavidin plates, and adhesive sealed plates were incubated for 1 hour with shaking at 700 rpm at 25 °C on an orbital shaker (Jitterbug-2, Boekel Scientific, Feasterville, PA, USA). The plates were washed three times with 150 μL/well of wash buffer (0.01 M PBS pH 7.4 with 0.05% Tween-20) and tapped on absorbent Kimwipes (Catalog number 34133, Kimberly-Clark Global Sales, LLC, Roswell, GA, USA). MSD supplied human plasma-purified APOE calibrator standard concentrations (750, 187.50, 46.88, 11.72, 2.93, 0.73, 0.18 ng/mL) were prepared in the assay diluent 57 (Catalog number R50BZ, MSD). The assay diluent was used as the zero-calibrator standard. Human EDTA plasma samples were diluted 100-fold, and CSF samples were diluted 50-fold in the assay diluent 57 in protein LoBind tubes (Eppendorf AG, Hamburg, Germany). Twenty-five microliters of diluent 57 was added to each well of the coated plates prior to the addition of 25 μL of prepared calibrator standards or samples in designated wells. The plates were sealed and incubated for 1 hour with shaking at 700 rpm at 25° C on an orbital shaker. The plates were washed three times with 150 μL/well of wash buffer and tapped on absorbent Kimwipes to remove residual wash buffer. The supplied SULFO-TAGTM-conjugated 100X detection antibody was diluted in diluent 3 (Catalog number R50AP-2, MSD). Fifty microliters of the detection antibody solution were added to each well, and the sealed plates were incubated for 1 hour with shaking at 700 rpm at 25 °C on an orbital shaker. The plates were washed three times with 150 μL/well of wash buffer and tapped on absorbent Kimwipes to remove residual wash buffer. MSD read buffer T (Catalog number R92TC-2, MSD) was diluted 2-fold in UltraPure^™^ distilled water (Catalog number 10977–015, Invitrogen, Grand Island, NY, USA), and 150 μL of read buffer was added to each well. The plates were read immediately on an MESO Quickplex^®^ SQ120MM instrument with electrochemiluminescence detection. Calibrator standard curves were used to calculate plasma and CSF APOE concentrations, after correcting for the sample dilution factor.

### APOE3 and APOE4 Measurement in CSF and Plasma

CSF and plasma samples from baseline assessments were analyzed using an APOE mass spectrometric immunoassay (MSIA) as described previously (10). The MSIA detects 3 APOE isoforms at the intact protein level, with apoE affinity-captured from CSF and plasma using MSIA tips derivatized with APOE-specific antibodies. A MALDI-TOF mass spectrometer (Autoflex III MALDI-TOF, Bruker, Billerica, MA) was used to analyzed eluted and intact APOE in positive ion mode, with a mass range of 7–70 kDa, 700 ns delay, 20.00 kV and 18.45 kV ion source voltages, and < 7000 Da signal suppression. Mass spectra were baseline subtracted (Convex Hull algorithm with 0.8 flatness) and smoothed (Savizky Golay algorithm with 5 m/z width and 1 cycle) using the Bruker Flex Analysis software. Zebra 1.0 software (Intrinsic Bioprobes Inc.) was used to quantify and tabulate the peak intensities of *APOE* isoforms. A variable representative of relative abundance of ApoE4 to ApoE3 in heterozygotes (ε4/ε3-ratio) was calculated by dividing CSF ApoE4 isoform level by CSF ApoE3 isoform level. An additional variable (percent ε4) was derived by multiplying total CSF ApoE4 isoform proportion by the CSF total ApoE concentration, representative of absolute concentration of ApoE4 protein in CSF (μg/mL).

### Small and Large HDL Measurement

Concentrations of HDL particles were measured by ion mobility (IM) after treatment with dextran sulfate to remove non-lipid-bound proteins such as albumin from 30 μL of plasma or CSF as described previously (14). Additional details can be found in the Supplementary Materials (7). Particles in the HDL size range were classified as small (7.0–10.5 nm) and large (10.5–14.5 nm) HDL. The majority of HDL particles in both CSF and plasma were found in the small size range, with a greater proportion of small to large HDLs present in CSF compared to plasma (7).

### Statistical Analysis

In cross-sectional analyses, linear regression models adjusting for age, sex, education, ε4 positivity, and clinical group were used to examine the associations between baseline independent variables (CSF and plasma small and large HDL/LDL particles, percent ε4, and ε4/ε3-ratio) and AD biomarker outcomes. Pearson’s correlations were used to examine the linear association of these independent variables measured in plasma vs CSF.

To evaluate between-group differences on CSF HDL levels and APOE concentrations, we utilized one-way analysis of variance (ANOVA) tests, followed by post-hoc pairwise comparisons using Tukey’s honestly significant difference (HSD) test to control for multiple comparisons. Group comparisons were made on *APOE* genotype groups (using ANOVA) as well as the two cognitive progression groups (CN vs MCI; Non-progressors vs Progressors) and CN vs MCI (using independent two-sample t-test).

Associations between baseline measures of small and large HDL particles and percent ε4 with longitudinally-assessed CSF tau, p-tau181, Aβ1–42 and cognitive performance annually over a 6-year period were tested using linear mixed effects models. HDL particle levels and fluid biomarker measures were log-transformed (log10(x + 1)) to reduce skew and improve normality, and all continuous variables were z-scored with a mean of 0 and SD of 1 to derive standardized parameter estimates. Fixed effects of interest were the baseline HDL and ε4 measures; adjusting covariates were age, sex, education, clinical group, and ε4 positivity (in models that did not include ε4 ratio or amount as an independent variable). A random participant-level intercept was specified to account for the within-participant correlation. We tested for an interaction between time modeled as a continuous variable (calculated as years since baseline assessment) and each of our independent variables to test for associations of baseline measures with longitudinal trajectories of change in outcome variables. Time was modeled as a fixed effect as well as a random participant-specific slope to account for longitudinal individual variation, and an unstructured variance-covariance matrix was specified for random effects. Likelihood ratio tests were conducted to compare models both with and without the addition of random slope to evaluate whether its inclusion was justified. We corrected for multiple comparisons across AD biomarker outcomes (Aβ1–42, tau, p-tau181) using the false discovery rate (FDR) method. All analyses used RStudio (Version 2024.12.1 + 563); the ‘lme4’ package was used for linear mixed effect modeling.

## Results

### HDL Subclasses and APOE Levels on Clinical Outcomes

**Figure 1:**
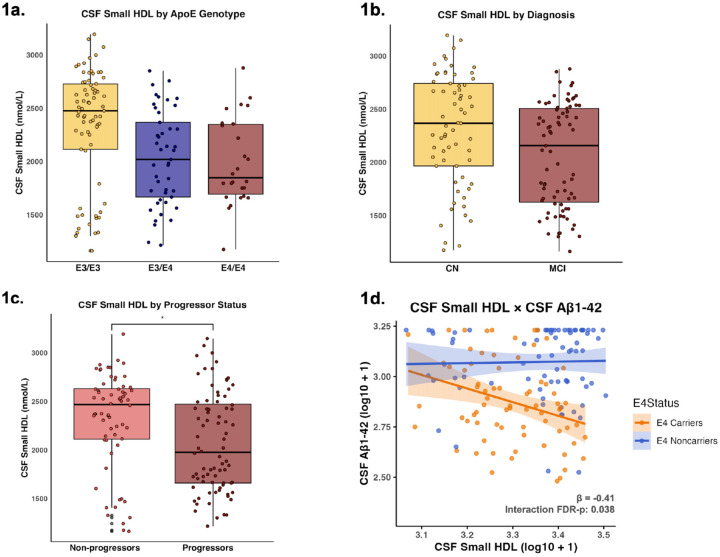
Small HDL as a function of Clinical Stage AD Biomarkers

CSF small HDL levels were significantly lower in ε3/ε4 genotypes (β = −0.06; p = 0.017) and in ε4/ε4 genotypes (β = −0.06; p = 0.033) compared to ε3/ε3 genotypes (Fig. 1a). No significant difference was observed between ε3/ε4 and ε4/ε4 genotypes (β = −0.01; p = 0.974). CN individuals had significantly higher levels of CSF small HDL compared to those with MCI (β = 0.05; p = 0.006) (Fig. 1b), as did cognitive non-progressors compared to progressors (β = 0.05; p = 0.010) (Fig. 1c).

**Figure 2:**
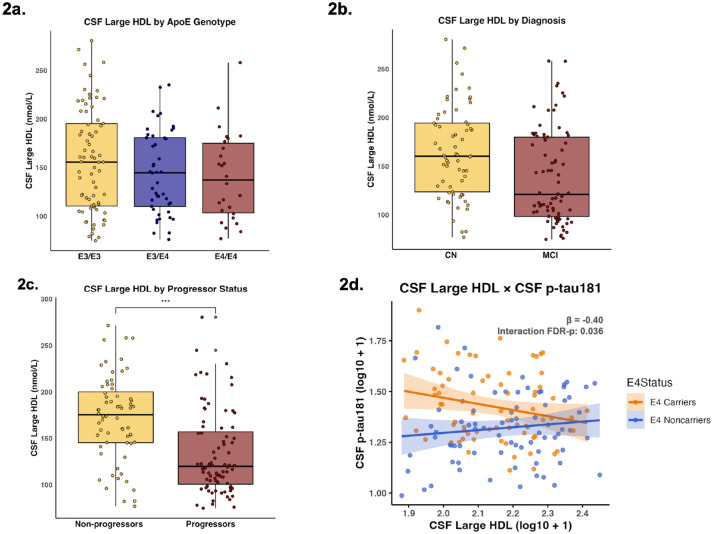
Large HDL as a function of Clinical Stage AD Biomarkers

CSF large HDL levels did not significantly differ across APOE genotypes (ε3/ε3 vs ε3/ε4: β = −0.03, p = 0.497; ε3/ε3 vs ε4/ε4: β = −0.05, p = 0.348; ε3/ε4 vs ε4/ε4: β = −0.01, p = 0.907) (Fig. 2a). CSF large HDL did significantly differ across cognitive diagnoses and progressor status, with higher levels of large HDL in CN (β = 0.08; p = 0.001) compared to MCI (Fig. 2b), and higher levels in non-progressors compared to progressors (β = 0.11 p < 0.001) (Fig. 2c).

**Figure 3:**
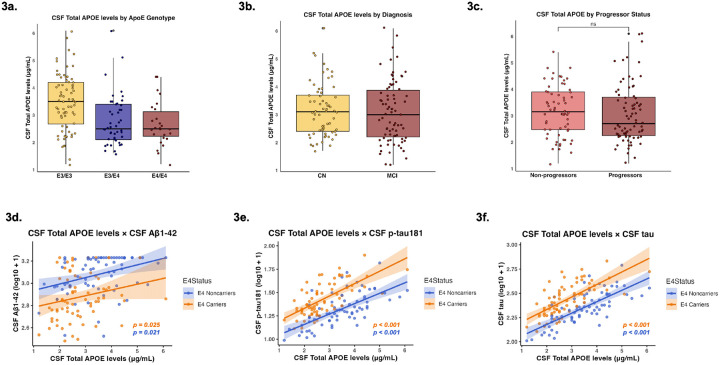
CSF Total APOE as a function of Clinical Stage AD Biomarkers

CSF total APOE levels significantly differed by genotype, with lower CSF total APOE levels in ε3/ε4 (β = −0.69; p < 0.001) and ε4/ε4 genotypes (β = −0.80; p = 0.001), compared to ε3/ε3 genotypes (Fig. 3a). Total APOE levels in CSF did not differ significantly between cognitive diagnoses (β = 0.06; p = 0.730) (Fig. 3b). Total APOE levels in CSF also did not significantly differ by progressor status in CSF (β = 0.11; p = 0.541) (Fig. 3c).

**Figure 4:**
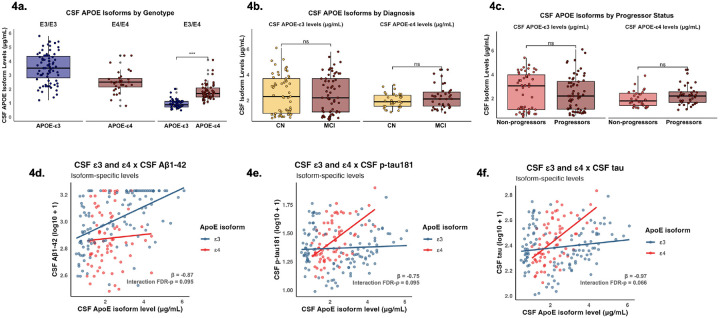
CSF APOE isoform concentrations as a function of Clinical Stage and AD Biomarkers

CSF APOE-ε3 levels were significantly higher in ε3/ε3 genotypes than ε3/ε4 (β = 2.53; p < 0.001) (Fig. 4a), but APOE-ε3 levels did not significantly differ across cognitive diagnoses (β = −0.03; p = 0.912) (Fig. 4b) or progressor status groups (β = 0.20; p = 0.463) (Fig. 4c). CSF APOE-ε4 levels were significantly higher in ε4/ε4 genotypes than ε3/ε4 (β = 0.79; p < 0.001) (Fig. 4a), but APOE-ε4 levels did not significantly differ across cognitive diagnoses (β = −0.27; p = 0.125) (Fig. 4b) or progressor status groups (β = −0.33; p = 0.073) (Fig. 4c). In ε3/ε4 heterozygous carriers, CSF APOE-ε4 levels were significantly higher than CSF APOE-ε3 levels (β = 0.89; p < 0.001) (Fig. 4a).

### APOE Isoform Levels, APOE-ε4/ε3 ratio, and APOE-ε4% in CSF

There was a trend-level interaction between CSF APOE-ε3 levels and CSF APOE-ε4 levels with baseline CSF Aβ1–42 (β = −0.87; p = 0.076; FDR-p = 0.095) (Fig. 4d) and CSF p-tau181 (β = −0.75; p = 0.095; FDR-p = 0.095) (Fig. 4e); however, associations were not sustained following FDR correction. There was a significant interaction between CSF APOE-ε3 levels and CSF APOE-ε4 levels with baseline CSF tau (β = −0.97; p = 0.022; FDR-p = 0.066) that became trend-level following FDR correction (Fig. 4f).

**Figure 5:**
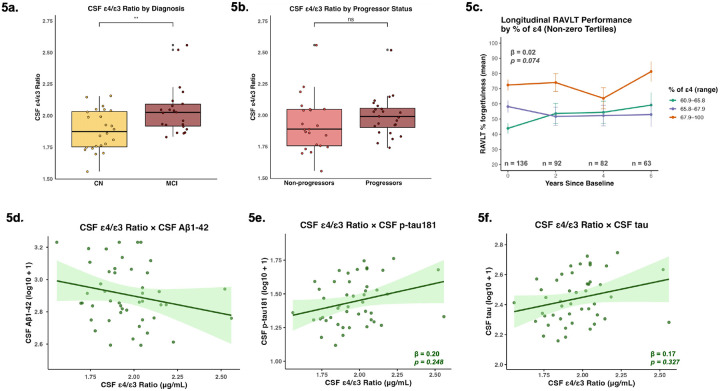
CSF ε4/ε3 Ratio as a function of Clinical Stage and AD Biomarkers

The CSF ε4/ε3 ratio was significantly higher in those with MCI compared to CN (β = 0.16; p = 0.004) (Fig. 5a) and did not significantly differ between cognitive progressors and non-progressors (β = −0.07; p = 0.256) (Fig. 5b). Cross-sectional associations between the CSF ε4/ε3 ratio and CSF Aβ1–42 (β = −0.05; p = 0.742), CSF p-tau181 (β = 0.20; p = 0.248), and CSF tau (β = 0.17; p = 0.327) were not significant (Fig. 5d–f).

### Clinical Outcome Differences Across APOE Levels and Ratios

Total plasma APOE levels (in μg/mL) differed by genotype, with lower plasma total APOE in ε3/ε4 genotypes (β = −7.59; p < 0.001) and ε4/ε4 genotypes (β = −7.16; p < 0.001), relative to ε3/ε3 genotypes (**Supplementary Fig. 1a**). Total plasma APOE levels did not differ significantly by cognitive diagnoses (β = −1.16; p = 0.343) (**Supplementary Fig. 1b**) or progressor status in plasma (β = −0.53; p = 0.653) (**Supplementary Fig. 1c**).

Plasma APOE-ε4 levels were significantly higher in homozygous carriers compared to heterozygous carriers (β = 1.47; p = 0.009) (**Supplementary Fig. 2a**) and in MCI compared to CN (β = 1.04; p = 0.023) (**Supplementary Fig. 2b**), but did not significantly differ by progressor groups (β = 0.07; p = 0.890) (**Supplementary Fig. 2c**).

### Cross-Sectional Associations: CSF HDLs and CSF APOE levels with CSF Biomarkers

APOE-ε4 carrier status significantly moderated the relationship between CSF small HDL and CSF Aβ1–42 (interaction β = −0.41; p = 0.025; FDR-p = 0.038), such that small HDL was significantly associated with lower CSF Aβ1–42 in APOE-ε4 carriers (β = −0.45; p = 0.001) and not in non-carriers (β = −0.04; p = 0.687) (Fig. 1d) (Table 2). APOE-ε4 carrier status also moderated the relationship between CSF small HDL and CSF p-tau181 at a trend-level (interaction β = −0.40; p = 0.054; FDR-p = 0.054), such that small HDL was at a trend-level associated with higher CSF p-tau181 among non-carriers (β = 0.21; p = 0.076), whereas no significant association was observed among carriers (β = −0.19; p = 0.229) (Table 2). APOE-ε4 carrier status furthermore significantly moderated the relationship between CSF small HDL on CSF tau (β = −0.52, p = 0.015; FDR-p = 0.038), with higher levels of small HDL trend-level associated with higher levels of CSF tau in non-carriers (β = 0.23; p = 0.058) but oppositely associated with lower levels of CSF tau in carriers (β = −0.29; p = 0.073) (Table 2).

APOE-ε4 significantly moderated the association between CSF large HDL and CSF p-tau181 (β = −0.40; p = 0.024; FDR-p = 0.036), with a non-significant positive trend in APOE-ε4 non carriers (β = 0.19, p = 0.110) and a non-significant negative trend in carriers (β = −0.20, p = 0.143) (Table 2). APOE-ε4 significantly moderated the association between CSF large HDL and CSF tau (β = −0.52; p = 0.003; FDR-p = 0.009), such that in APOE-ε4 non-carriers, higher CSF large HDL was significantly associated with higher CSF tau (β = 0.24, p = 0.049) but with lower CSF tau in APOE-ε4 carriers (β = −0.28, p = 0.046) (Table 2). We observed a trend-level interaction between APOE-ε4 x CSF large HDL with CSF Aβ1–42 (β = −0.28; p = 0.071; FDR-p = 0.071), with APOE-ε4 non-carriers having no significant association with CSF Aβ1–42 (β = 0.06, p = 0.576) and APOE-ε4 carriers having a trend-level association between higher CSF large HDL with lower CSF Aβ1–42 (β = −0.22, p = 0.075) (Table 2).

In the whole sample, CSF total APOE was cross-sectionally associated with significantly higher levels of CSF Aβ1–42 (β = 0.27; p = 0.007; FDR-p = 0.007) (Fig. 3d), CSF p-tau181 (β = 0.66; p < 0.001; FDR-p < 0.001) (Fig. 3e), and CSF tau (β = 0.74; p < 0.001; FDR-p < 0.001) (Fig. 3f) (Table 3). Plasma total APOE did not significantly associate with CSF Aβ1–42 (β = −0.01; p = 0.890; FDR-p = 0.890) (**Supplementary Fig. 1d**), CSF p-tau181 (β = 0.04; p = 0.711; FDR-p = 0.890) (**Supplementary Fig. 1e**), or CSF tau (β = 0.03; p = 0.788; FDR-p = 0.890) (**Supplementary Fig. 1f**) at baseline (Table 3). Total APOE levels were not associated with delayed memory in both CSF (β = 0.08; p = 0.436) and plasma (β = −0.17; p = 0.092) (Table 3).

Higher levels of CSF APOE-ε4 were not significantly associated with higher levels of CSF Aβ1–42 (β = 0.09; p = 0.422; FDR-p = 0.422) but were significantly associated with higher levels of both CSF p-tau181 (β = 0.48; p < 0.001; FDR-p < 0.001) and CSF tau (β = 0.49; p < 0.001; FDR-p < 0.001) (Table 3). CSF APOE-ε4 was not significantly associated with delayed memory (β = 0.15; p = 0.142) (Table 3). Higher levels of plasma APOE-ε4 were not significantly associated with CSF Aβ1–42 (β = −0.18; p = 0.124; FDR-p = 0.372) (**Supplementary Fig. 2d**), CSF p-tau181 (β = 0.003; p = 0.978; FDR-p = 0.978) (**Supplementary Fig. 2e**), CSF tau (β = −0.05; p = 0.685; FDR-p = 0.978) (**Supplementary Fig. 2f**) or delayed memory (β = −0.02; p = 0.884) at baseline (Table 3).

### Longitudinal Associations

We observed a trend-level longitudinal relationship between baseline CSF percent ε4 and longitudinal changes in delayed memory, such that a higher baseline percentage of ε4 trended with poorer memory recall over a 6-year period (β = 0.02; p = 0.074) (Fig. 5c). Baseline measurements of CSF percent ε4 were not significantly associated with CSF Aβ1–42 (β = −0.02; p = 0.140), CSF p-tau181 (β = −0.01; p = 0.417), or CSF tau (β = −0.01; p = 0.463) longitudinally. We additionally observed trend-level relationships between baseline measurements of both CSF small HDL and CSF large HDL with CSF p-tau181 longitudinally, but these associations were not maintained following FDR-p correction (β = 0.01; p = 0.069; FDR-p = 0.208 for CSF small HDL; β = 0.01; p = 0.063; FDR-p = 0.190 for CSF large HDL). No associations were observed between baseline measurements of CSF small HDL with longitudinal CSF Aβ1–42 (β = −0.0001; p = 0.985; FDR-p = 0.985) or CSF tau (β = 0.01; p = 0.216; FDR-p = 0.324), or between baseline measurements of CSF large HDL with longitudinal CSF Aβ1–42 (β = 0.001; p = 0.876; FDR-p = 0.876) or CSF tau (β = 0.01; p = 0.307; FDR-p = 0.460).

## Discussion

In this study, we analyzed HDL particle concentrations together with total and isoform-specific APOE protein levels in CSF and plasma and examined how they relate to CSF AD biomarkers in a well-characterized ADNI cohort. Our findings indicate that CNS, rather than peripheral, APOE4 measures align more closely with AD-related biomarkers. Specifically, higher CSF APOE4 protein levels were associated with higher CSF tau and p-tau181 concentrations, whereas plasma APOE4 measures showed minimal and inconsistent relationships with CSF-measured pathology. In addition, the CSF ε4/ε3 ratio was elevated in MCI and in individuals with cognitive progression, while plasma patterns differed in direction, underscoring compartment-specific APOE biology.

These results add important nuance to the small HDL hypothesis of AD (7). While that hypothesis emphasizes the neuroprotective capacity of small HDL particles, through ABCA1-mediated cholesterol efflux, lipid exchange, and synaptic maintenance, our data suggest that this protective biology is insufficient to counteract APOE4 protein burden in the CNS. CSF small HDL correlated positively with ABCA1-mediated cholesterol efflux ex vivo (15) and with better cognition in an earlier cohort (7), yet failed to buffer APOE4-biomarker associations in these current analyses. This implies that the pathogenic effects of APOE4 may act through mechanisms at least partially independent of HDL-mediated lipid transport, such as direct promotion of tau seeding, neuroinflammation, or impaired receptor-mediated clearance. The compartment specificity of our findings further aligns with evidence that CSF small HDL formation shares common APOE-dependent mechanisms rather than reflecting passive transport from plasma (16). Consistent with prior reports (7), APOE4 carriers exhibited reduced CSF small HDL, yet higher HDL particle levels did not attenuate APOE4-biomarker associations. APOE4 status continued to moderate relationships with Aβ1–42, total tau, and p-tau181 independent of HDL subclass distribution, arguing against a lipidation-deficiency model and instead suggesting that APOE4 protein burden itself drives biomarker associations.

The opposing directions of HDL-biomarker associations across genotype groups warrant consideration. In APOE4 non-carriers, the positive association between CSF small HDL and tau may reflect reactive upregulation of HDL production in response to early neurodegeneration, as has been hypothesized for total CSF APOE (8). In carriers, the inverse association could represent residual ABCA1 function: individuals who maintain higher small HDL despite the ε4 allele may retain better APOE lipidation and less APOE4 aggregation (17). However, this residual protective signal was overwhelmed by the dominant association between APOE4 protein levels and tau. These genotype-dependent directional differences underscore that HDL particle measurements cannot be interpreted uniformly across *APOE* genotype groups and suggest that the functional relationship between HDL and neurodegeneration is fundamentally reconfigured by the presence of APOE4.

The divergence between plasma and CSF findings further emphasizes the importance of compartmentalization. Plasma APOE measures did not consistently reflect central biomarker status and in some analyses demonstrated opposing patterns, indicating that peripheral APOE dynamics may not mirror CNS APOE biology. Systemic lipid measures are therefore insufficient proxies for central disease mechanisms, and therapeutic strategies aimed at modifying peripheral HDL or systemic APOE may have limited impact on CNS pathology.

Analyses within ε3/ε4 heterozygotes reveal that APOE4 burden behaves in a graded manner, scaling with relative ε4 protein proportion rather than genotype status alone. Higher baseline CSF ε4 proportion trended toward greater longitudinal memory decline over six years, supporting a dose-like relationship between CNS APOE4 burden and cognitive vulnerability (18). Although the association was trend-level and this study does not establish a specific reduction threshold, these graded associations suggest that partial lowering of CNS APOE4 may shift the biomarker profile toward a less pathogenic state. The absence of concurrent associations between CSF percent ε4 and longitudinal AD biomarkers is suggestive of alternate pathways, independent of amyloid and tau, that might mediate APOE4-associated effects on memory.

This dose-response pattern provides human evidence aligned with a growing consensus that reducing CNS APOE4 should be a therapeutic goal. An NIA/ADSP Consortium Working Group concluded that 50% reduction in CNS APOE4 is likely tolerable and therapeutic (19). Several programs are now testing this premise: LX1001, an AAV-delivered APOE2 gene therapy for APOE4 homozygotes, produced dose-dependent APOE2 expression in CSF and reductions in tau biomarkers in Phase 1/2 (20); preclinical APOE4-to-APOE2 allelic switching in astrocytes reduced amyloid pathology and restored cognition in 5xFAD mice (21); APOE-targeted ASOs achieved approximately 50% CNS expression reduction with attenuation of tau pathology and microglial activation (22); and focused ultrasound-mediated CRISPR delivery has been demonstrated for brain-targeted APOE4 knockdown (23). Our data complement these efforts by demonstrating that quantitative CSF APOE4 protein levels, not genotype status alone, track with tau and p-tau181 in humans, suggesting that CSF APOE4 measurement could serve as a pharmacodynamic biomarker for APOE4-lowering trials. An additional consideration is whether APOE4 modifies the therapeutic window for tau-directed interventions. The anti-tau antibody bepranemab showed reduced tau-PET accumulation preferentially in APOE4 non-carriers with low baseline tau (24), suggesting that APOE4 may narrow the window during which tau-targeted therapies are effective. This raises the possibility that combination strategies, pairing APOE4 reduction with tau-targeted therapy, may be needed for carriers.

This study has several limitations. Although the cohort included 144 participants with comprehensive biomarker characterization, subgroup analyses were performed in smaller subsets, which may limit statistical power and increase the risk of type II error. Moderation analyses examining APOE4-by-HDL interactions are inherently power-sensitive, and some interaction effects did not reach statistical significance in stratified models, warranting cautious interpretation. The ADNI cohort is predominantly White and highly educated, limiting generalizability to more diverse populations. The observational design precludes causal inference. Although HDL particle subclasses were quantified by size, we did not directly measure APOE content within specific HDL particles or assess APOE-containing HDL complexes, and therefore cannot determine whether APOE4 preferentially associates with particular HDL subspecies or whether functional lipidation state, rather than particle size, influences pathogenicity. Finally, we did not evaluate other lipoprotein classes, lipid species, or cell-specific APOE expression patterns, leaving unresolved whether astrocytic, microglial, or other CNS-derived APOE sources differentially contribute to the observed associations.

In conclusion, our findings support a CNS-compartment model in which APOE4 protein burden aligns more closely with tau pathology and cognitive decline than peripheral measures or HDL subclass distribution. The absence of a buffering effect of HDL particle abundance argues against lipidation-enhancing strategies as a primary therapeutic approach. Instead, these data identify CNS APOE4 burden as a biologically relevant and potentially modifiable target, and provide a framework for evaluating APOE4-directed interventions, including gene therapies, antisense oligonucleotides, and combination approaches with tau-targeted agents, in future clinical studies.

## Supplementary Material

Supplementary Files

This is a list of supplementary files associated with this preprint. Click to download.

- Table1Demographics.docx
- Table2APOE4CarrierStatusModerationofHDLBiomarkerAssociations.docx
- Table3CrossSectionalAnalyses.docx
- FinalHDLManuscriptSuppFigure104072026.png
- FinalHDLManuscriptSuppFigure204072026.png

Tables are available in the Supplementary Files section.

## Figures and Tables

**Table 1:** Demographic breakdown of participants with complete data for all covariates included at baseline.

N (baseline)	Whole Cohort	Cognitively Normal, NP	Cognitively Normal, P	Mild Cognitive Impairment, NP	Mild Cognitive Impairment, P
	144	27	38	37	42
**Age (mean ± SD)**	73.0 ± 6.6	73.9 ± 7.0	75.1 ± 5.2	71.2 ± 6.6	72.2 ± 7.1
**Sex (N, %)**	82 M (56.9%)	15 M (55.6%)	15 M (39.5%)	25 M (67.6%)	27 M (64.3%)
**APOE genotype (N, %)**					
*3/ 3*	73 (50.7%)	13 (48.1%)	20 (52.6%)	19 (51.4%)	21 (50.0%)
*3/ 4*	45 (31.3%)	11 (40.7%)	13 (34.2%)	11 (29.7%)	10 (23.8%)
*4/ 4*	26 (18.1%)	3 (11.1%)	5 (13.2%)	7 (18.9%)	11 (26.2%)
**Race (N, %)**					
*Asian*	2 (1.4%)	0 (0.0%)	0 (0.0%)	1 (2.7%)	1 (2.4%)
*Black*	7 (4.9%)	2 (7.4%)	1 (2.6%)	2 (5.4%)	2 (4.8%)
*White*	134 (93.1%)	25 (92.6%)	37 (97.4%)	34 (91.9%)	38 (90.5%)
*Other / Unknown*	1 (0.7%)	0 (0.0%)	0 (0.0%)	0 (0.0%)	1 (2.4%)
**Years of Education (mean ± SD)**	16.3 ± 2.7	16.2 ± 2.9	16.2 ± 2.6	16.9 ± 2.5	15.8 ± 2.6
**CSF Biomarkers (mean ± SD)**					
*Aβ1–42 (pg/mL)*	1060.9 ± 466.9	1231.0 ± 406.8	1114.5 ± 499.5	1025.0 ± 507.8	934.8 ± 405.8
*t-tau (pg/mL)*	263.7 ± 109.4	248.8 ± 67.9	249.9 ± 92.2	255.5 ± 104.4	293.2 ± 142.5
*p-tau181 (pg/mL)*	24.9 ± 12.0	22.8 ± 7.3	23.4 ± 9.5	24.1 ± 11.2	28.4 ± 16.0
**Small HDL (nmol/L) (mean ± SD)**	2177.0 ± 515.8	2360.3 ± 518.0	2282.9 ± 531.2	2272.0 ± 496.4	1879.6 ± 394.4
**Large HDL(nmol/L) (mean ± SD)**	150.2 ± 49.0	178.8 ± 44.9	154.1 ± 46.0	166.5 ± 46.6	114.0 ± 33.7

* NP = Non-Progressors, P=Progressors.

**Table 2:** APOE-ε4 carrier status moderation of HDL-biomarker associations. All regression models included covariates for age, sex, education, and clinical group.

CSF Small HDL x APOE- 4 on Aβ1–42	Interaction term (β, p-value, FDR-p)	APOE- 4 non-carrier slope (β, p-value)	APOE- 4 carrier slope (β, p-value)
	β = −0.41; p = 0.025; FDR-p = 0.038	β = −0.04; p = 0.687	β = −0.45; p = 0.001
**CSF Small HDL x APOE- 4 on tau**	β = −0.52; p = 0.015; FDR-p = 0.038	β = 0.23; p = 0.058	β = −0.29; p = 0.073
**CSF Small HDL x APOE- 4 on p-tau181**	β = −0.40; p = 0.054; FDR-p = 0.054	β = 0.21; p = 0.076	β = −0.19; p = 0.229
**CSF Large HDL x APOE- 4 on Aβ1–42**	β = −0.28; p = 0.071; FDR-p = 0.071	β = 0.06; p = 0.576	β = −0.22; p = 0.075
**CSF Large HDL x APOE- 4 on tau**	β = −0.52; p = 0.003; FDR-p = 0.009	β = 0.24; p = 0.049	β = −0.28; p = 0.046
**CSF Large HDL x APOE- 4 on p-tau181**	β = −0.40; p = 0.024; FDR-p = 0.036	β = 0.19; p = 0.110	β = −0.20; p = 0.143

**Table 3:** Cross-sectional results of CSF and plasma HDL levels, APOE, and APOE-ε4 measures with clinical biomarker and cognitive outcomes. All regression models included covariates for age, sex, education, clinical group, and ε4 positivity (in models that did not include ε4 ratio or amount as an independent variable).

CSF Total APOE	CSF Aβ1–42 (β, p-value, FDR- P)	CSF total tau (β, p-value, FDR-P)	CSF p-tau181 (β, p-value, FDR-p)	RAVLT % forgetfulness (β, p-value)
	β = 0.27; p = 0.007; FDR-p = 0.007	β = 0.74; p < 0.001; FDR-p< 0.001	β = 0.66; p < 0.001; FDR-p< 0.001	β = 0.08; p = 0.436
**Plasma Total APOE**	β = −0.01; p = 0.890; FDR-p = 0.890	β = 0.03; p = 0.788; FDR-p = 0.890	β = 0.04; p = 0.711; FDR-p = 0.890	β = −0.17; p = 0.092
**CSF APOE- 4**	β = 0.09; p = 0.422; FDR-p = 0.422	β = 0.49; p < 0.001; FDR-p < 0.001	β = 0.48; p < 0.001; FDR-p < 0.001	β = 0.15; p = 0.142
**Plasma Total APOE- 4**	β = −0.18; p = 0.124; FDR-p = 0.372	β = −0.05; p = 0.685; FDR-p = 0.978	β = 0.003; p = 0.978; FDR-p = 0.978	β = −0.02; p = 0.884

## References

[1] Yassine HN, Hugo C, O’Donovan B, Stephens IO, Johnson LA, Cole G APOE-Targeted Therapeutics for Alzheimer’s Disease. J Neurosci. 2025;45(46). 10.1523/JNEUROSCI.1388-25.2025 PMC12614056 41224653

[2] Di Battista AM, Heinsinger NM, Rebeck GW. Alzheimer’s Disease Genetic Risk Factor APOE-ε4 Also Affects Normal Brain Function. Curr Alzheimer Res. 2016;13(11):1200–7. 27033053 10.2174/1567205013666160401115127 PMC5839141

[3] Baek MS, Cho H, Lee HS, Lee JH, Ryu YH, Lyoo CH. Effect of APOE ε4 genotype on amyloid-β and tau accumulation in Alzheimer’s disease. Alzheimers Res Ther. 2020;12(1):140. 33129364 10.1186/s13195-020-00710-6 PMC7603688

[4] Liu M, Kuhel DG, Shen L, Hui DY, Woods SC. Apolipoprotein E does not cross the blood-cerebrospinal fluid barrier, as revealed by an improved technique for sampling CSF from mice. Am J Physiol Regul Integr Comp Physiol. 2012;303(9):R903–8. 22933021 10.1152/ajpregu.00219.2012 PMC3517701

[5] Linton MF, Gish R, Hubl ST, Bütler E, Esquivel C, Bry WI, Phenotypes of apolipoprotein B and apolipoprotein E after liver transplantation. J Clin Invest. 1991;88(1):270–81. 2056122 10.1172/JCI115288 PMC296029

[6] Fisher EA, Feig JE, Hewing B, Hazen SL, Smith JD. High-density lipoprotein function, dysfunction, and reverse cholesterol transport. Arterioscler Thromb Vasc Biol. 2012;32(12):2813–20. 23152494 10.1161/ATVBAHA.112.300133 PMC3501261

[7] Martinez AE, Weissberger G, Kuklenyik Z, He X, Meuret C, Parekh T, The small HDL particle hypothesis of Alzheimer’s disease. Alzheimers Dement. 2023;19(2):391–404. 35416404 10.1002/alz.12649 PMC10563117

[8] Martínez-Morillo E, Hansson O, Atagi Y, Bu G, Minthon L, Diamandis EP, Total apolipoprotein E levels and specific isoform composition in cerebrospinal fluid and plasma from Alzheimer’s disease patients and controls. Acta Neuropathol. 2014;127(5):633–43. 24633805 10.1007/s00401-014-1266-2

[9] Turri M, Conti E, Pavanello C, Gastoldi F, Palumbo M, Bernini F, Plasma and cerebrospinal fluid cholesterol esterification is hampered in Alzheimer’s disease. Alzheimers Res Ther. 2023;15(1):95. 37210544 10.1186/s13195-023-01241-6 PMC10199596

[10] Nedelkov D, Tsokolas ZE, Rodrigues MS, Sible I, Han SD, Kerman BE, Increased cerebrospinal fluid and plasma apoE glycosylation is associated with reduced levels of Alzheimer’s disease biomarkers. Alzheimers Res Ther. 2025;17(1):151. 40635030 10.1186/s13195-025-01795-7 PMC12243426

[11] Olsson A, Vanderstichele H, Andreasen N, De Meyer G, Wallin A, Holmberg B, Simultaneous measurement of beta-amyloid(1–42), total tau, and phosphorylated tau (Thr181) in cerebrospinal fluid by the xMAP technology. Clin Chem. 2005;51(2):336–45. 15563479 10.1373/clinchem.2004.039347

[12] Tosun D, Hausle Z, Thropp P, Concha-Marambio L, Lamoureux J, Lebovitz R, Association of CSF α-synuclein seed amplification assay positivity with disease progression and cognitive decline: A longitudinal Alzheimer’s Disease Neuroimaging Initiative study. Alzheimers Dement. 2024;20(12):8444–60. 39428831 10.1002/alz.14276 PMC11667524

[13] Crane PK, Carle A, Gibbons LE, Insel P, Mackin RS, Gross A, Development and assessment of a composite score for memory in the Alzheimer’s Disease Neuroimaging Initiative (ADNI). Brain Imaging Behav. 2012;6(4):502–16. 22782295 10.1007/s11682-012-9186-z PMC3806057

[14] Mora S, Caulfield MP, Wohlgemuth J, Chen Z, Superko HR, Rowland CM, Atherogenic Lipoprotein Subfractions Determined by Ion Mobility and First Cardiovascular Events After Random Allocation to High-Intensity Statin or Placebo: The Justification for the Use of Statins in Prevention: An Intervention Trial Evaluating Rosuvastatin (JUPITER) Trial. Circulation. 2015;132(23):2220–9. 26408274 10.1161/CIRCULATIONAHA.115.016857 PMC4674425

[15] Yassine HN, Feng Q, Chiang J, Petrosspour LM, Fonteh AN, Chui HC ABCA1-Mediated Cholesterol Efflux Capacity to Cerebrospinal Fluid Is Reduced in Patients With Mild Cognitive Impairment and Alzheimer’s Disease. J Am Heart Assoc. 2016;5(2). 10.1161/JAHA.115.002886 PMC4802440 26873692

[16] Van Valkenburgh J, Meuret C, Martinez AE, Kodancha V, Solomon V, Chen K, Understanding the Exchange of Systemic HDL Particles Into the Brain and Vascular Cells Has Diagnostic and Therapeutic Implications for Neurodegenerative Diseases. Front Physiol. 2021;12:700847. 34552500 10.3389/fphys.2021.700847 PMC8450374

[17] Rawat V, Wang S, Sima J, Bar R, Liraz O, Gundimeda U, ApoE4 Alters ABCA1 Membrane Trafficking in Astrocytes. J Neurosci. 2019;39(48):9611–22. 31641056 10.1523/JNEUROSCI.1400-19.2019 PMC6880458

[18] Huang YA, Zhou B, Nabet AM, Wernig M, Südhof TC. Differential Signaling Mediated by ApoE2, ApoE3, and ApoE4 in Human Neurons Parallels Alzheimer’s Disease Risk. J Neurosci. 2019;39(37):7408–27. 31331998 10.1523/JNEUROSCI.2994-18.2019 PMC6759032

[19] Vance JM, Farrer LA, Huang Y, Cruchaga C, Hyman BT, Pericak-Vance MA, Report of the APOE4 National Institute on Aging/Alzheimer Disease Sequencing Project Consortium Working Group, Reducing APOE4 in Carriers is a Therapeutic Goal for Alzheimer’s Disease. Ann Neurol. 2024;95(4):625–34. 38180638 10.1002/ana.26864

[20] Johnson KG, Kaplitt M, Kaminsky S, Sondhi D, Amato G, Selvan N, Topline results from Phase 1/2 AAV gene therapy (LX1001) in APOE4/4 homozygotes with Alzheimer’s disease. Alzheimer’s Dement. 2025;21(S5):e101538.

[21] Golden LR, Siano DS, Stephens IO, MacLean SM, Saito K, Nolt GL, APOE4 to APOE2 allelic switching in mice improves Alzheimer’s disease-related metabolic signatures, neuropathology and cognition. Nat Neurosci. 2025;28(12):2461–75. 41219507 10.1038/s41593-025-02094-y PMC12672373

[22] Huynh TV, Liao F, Francis CM, Robinson GO, Serrano JR, Jiang H, Age-Dependent Effects of apoE Reduction Using Antisense Oligonucleotides in a Model of β-amyloidosis. Neuron. 2017;96(5):1013–e234. 29216448 10.1016/j.neuron.2017.11.014 PMC5728673

[23] Zheng K, Tsitsos FN, Batts AJ, Ji R, Nuriel T, Konofagou EE, Focused ultrasound-mediated APOE4 knockdown in mouse brain. Alzheimers Dement. 2025;21(7):e70464. 40665471 10.1002/alz.70464 PMC12263348

[24] Sidhu JS, Sardu ML, Björnsson MA, Gallais F, Khandelwal A, Byrnes W, Establishing bepranemab posology through exposure-response modeling and simulation for TOGETHER, a double-blind, placebo-controlled Phase II study of bepranemab in prodromal–mild Alzheimer’s disease (AD). Alzheimer’s Dement. 2025;21(S5):e099277.

